# Recombinant Protein Expression for Structural Biology in HEK 293F Suspension Cells: A Novel and Accessible Approach

**DOI:** 10.3791/51897

**Published:** 2014-10-16

**Authors:** Nicola Portolano, Peter J. Watson, Louise Fairall, Christopher J. Millard, Charles P. Milano, Yun Song, Shaun M. Cowley, John W.R. Schwabe

**Affiliations:** ^1^Department of Biochemistry, University of Leicester

**Keywords:** Biochemistry, Issue 92, structural biology, protein expression, recombinant protein, mammalian cell, transfection, polyethylenimine, suspension culture, affinity purification.

## Abstract

The expression and purification of large amounts of recombinant protein complexes is an essential requirement for structural biology studies. For over two decades, prokaryotic expression systems such as *E. coli* have dominated the scientific literature over costly and less efficient eukaryotic cell lines. Despite the clear advantage in terms of yields and costs of expressing recombinant proteins in bacteria, the absence of specific co-factors, chaperones and post-translational modifications may cause loss of function, mis-folding and can disrupt protein-protein interactions of certain eukaryotic multi-subunit complexes, surface receptors and secreted proteins. The use of mammalian cell expression systems can address these drawbacks since they provide a eukaryotic expression environment. However, low protein yields and high costs of such methods have until recently limited their use for structural biology. Here we describe a simple and accessible method for expressing and purifying milligram quantities of protein by performing transient transfections of suspension grown HEK (Human Embryonic Kidney) 293F cells.

**Figure:**
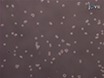


## Introduction

The rapid progress of molecular cell biology and the constant need for improved drugs in medicine has created a need for structural biologists to look at increasingly more complex protein structures. These can often require particular post-translational modifications, molecular chaperones and co-factors to support their elaborate folding and enzymatic activity. Whilst the vast majority of structures present in the Protein Data Bank have been obtained using bacterial expression systems, prokaryotes are unable to perform a large number of these modifications and lack many essential eukaryotic co-factors. This can be an issue for the study of large multi-subunit complexes that are activated by small signaling molecules as well as for nuclear, cell surface and secreted proteins that require elaborate folding machineries. A number of *E. coli* strains have been engineered to overcome some of these limitations^1^. In recent years, however, the use of mammalian expression systems has been increasing because they reliably produce eukaryotic proteins that are otherwise problematic to express in other systems^2^. Today it is possible to obtain stable and transient mammalian expression cell lines through a variety of techniques that range from viral transduction to chemical-mediated transfection, physical gene-transfer such as electroporation and direct injection^3^. While all of these methods have their own set of advantages and disadvantages, only a few of them are suitable for structural studies being either too expensive and/or time consuming.

Here we describe a very simple, fast, inexpensive yet highly efficient method for expressing protein complexes for structural biology in suspension grown mammalian cells. The approach uses transient co-transfection of a Human Embryonic Kidney (HEK) cell line (*e.g.,* Freestyle HEK 293F cells). These cells have been derived from the HEK 293 cell line, are adapted to grow in suspension cultures, reaching high densities using serum-free media (such as FreeStyle 293 Expression Medium). Cells are then transiently transfected using a branched version of polyethylenimine (PEI), an inexpensive polymeric reagent that has been reported to function for a large range of mammalian cells^4^ by forming DNA/PEI complexes that enter the host cell by endocytosis^5^. This method is suitable for both small scale (30 ml) and large-scale (up to 300 ml) experiments and can produce high levels of purified protein complexes. It is particularly useful for studying proteins that require complex folding machineries, co-factors or particular post-translational modifications that cannot be performed by bacteria, yeast and insect cells.

In this protocol we present the expression and purification of the three-protein central scaffold from the Sin3A transcriptional repression complex. This consists of Histone Deacetylase 1 (HDAC1), Suppressor of Defective Silencing 3 (SDS3) and Switch-independent 3 (Sin3A). The purified complex is used for high-throughput crystallization trials.

## Protocol

NOTE: The protocol is suitable for any scale of expression, therefore volumes and quantities of reagents should be scaled proportionally. A suitable mammalian expression vector must be used for this protocol. Here we used a modified pcDNA 3.1 expression vector that conveniently allows the inclusion or exclusion of an affinity tag depending on the choice of restriction enzymes used in the cloning (**Figure 1**). The following protocol describes a large scale transfection.

### 1. Large Scale Culture/Transfection in 1 L Roller Bottles

Grow and maintain HEK293F suspension-adapted cells according to standard protocols. Typically starter cultures of between 30 and 100 ml are grown in 250 ml conical cell culture flasks.

1. Seed cells at 0.5 x 10^6^ cells/ml into a final volume of 300 ml in each 1 L roller bottle. NOTE: Roller bottles accommodate a minimum of 150 ml to a maximum of 300 ml of suspension culture. For larger scale transfections use multiple bottles.
2. Incubate for 24 hr in an orbital shaker incubator at 37 °C, 120 rpm, and 5% CO_2_ until cells reach a density of 1.0 x 10^6^ cells/ml (cells should divide approximately every 24 hr).
3. Pipette a total of 300 µg of filter-sterilized DNA (see supplementary method) into 30 ml of PBS and vortex vigorously for 3 sec. NOTE: Use a total of 1 µg of DNA per million transfected cells. If two or more plasmids are to be co-transfected reduce the amount of each so that the ratio of DNA to cells remains constant. 
4. Add 1.2 ml of 0.5 mg/ml filter-sterilized PEI to the PBS/DNA solution and vortex vigorously for 3 sec.
5. Incubate the mix at RT for 20 min.
6. Add the DNA/PEI mix to the cells – which should be at a density of 1 x 10^6 ^ cells/ml (step 1.3).
7. Following co-transfection, incubate the cells in an orbital shaker incubator for a further 48 hr at 37 °C, 120 rpm, and 5% CO_2_.
8. Harvest intracellular proteins by centrifuging cells at 3,000 x g for 5 min and store the pellet at -80 °C. NOTE: Alternatively, use a standard (non-CO_2_) shaking incubator, if the atmosphere above the culture is replaced with 5% CO_2_ and the bottle lid is sealed. The atmosphere will need to be replaced at each passage (normally every 2 days).

### 2. Protein-complex Purification from Whole Cell Extract

This protocol is optimized for the purification of nuclear complexes using flag-tagged proteins.

1. Defrost cell pellet into ~40 ml of pre-chilled lysis buffer (100 mM potassium acetate, 50 mM Tris pH 7.5, 5% glycerol, 0.3% Triton X-100, protease inhibitors) per L of culture.
2. Re-suspend the pellet by pipetting up and down several times (to avoid foaming, do not vortex).
3. Thoroughly re-suspend cells using a glass homogenizer. Sonicate for 3 cycles (15 sec on, 15 sec off). Centrifuge at 30,000 x g for 25 min at 4ºC and retain supernatant.
4. Equilibrate 1.25 ml of anti-flag packed agarose resin per liter of culture by washing three times with resin equilibration buffer (100 mM potassium acetate, 50 mM Tris pH 7.5).
5. Incubate the whole cell extract from step 2.3 with the affinity resin in one or more 50 ml centrifuge tubes and gently rotate the sample for 30-120 min at 4 °C.
6. Centrifuge at 3,000 x g for 1 min at 4 °C, discard supernatant.
7. Wash the resin with 45 ml of pre-chilled buffer 1 (100 mM potassium acetate, 50 mM Tris pH 7.5, 5% glycerol, 0.3% Triton X-100). Centrifuge at 3,000 x g for 1 min and discard supernatant.
8. Repeat step 2.7 using high salt buffer (300 mM potassium acetate, 50 mM Tris pH 7.5, 5% glycerol), followed by low salt buffer (50 mM potassium acetate, 50 mM Tris pH 7.5, 5% glycerol) and TEV Cleavage Buffer (50 mM potassium acetate, 50 mM Tris pH 7.5, 0.5 mM TCEP). NOTE: Ensure each wash is brief* i.e.,* sufficient to fully re-suspend the resin and no longer.
9. Collect a 10 µl sample of resin and dilute into 1 volume of 2x protein loading buffer for analysis (bound protein control). Do not use reducing agents in order to avoid releasing the antibody from the resin.
10. Re-suspend the resin into 8-10 ml of pre-chilled TEV cleavage buffer and transfer to a 15 ml centrifuge tube.
11. Add ~40 µg of TEV protease (from stock at 1 mg/ml) per L of original culture and mix well by pipetting up and down several times.
12. Replace tube atmosphere with 100% N_2_ gas to prevent protein oxidation.
13. Gently rotate O/N at 4ºC.
14. Centrifuge at 3,000 x g for 10 min. Transfer the supernatant into an ultra centrifugal filter with an appropriate molecular weight cut-off and concentrate down to 500 µl.
15. Collect a 10 µl sample of the concentrated protein and dilute into 1 volume of 2x protein loading buffer for analysis. (TEV eluate control).
16. Collect a 10 µl sample of the resin and dilute into 1 volume of 2x protein loading buffer for analysis. Do not use reducing agents in order to avoid releasing large amounts antibody from the resin. (Post-TEV resin control).
17. Equilibrate the size exclusion chromatography column with gel filtration buffer (50 mM potassium acetate, 50 mM Tris pH 7.5, 0.5 mM TCEP).
18. Filter the protein through a 0.22 µm filter
19. Load the sample into the column and collect the fractions.
20. Run samples from steps 2.9, 2.15 and 2.16 and the gel filtration fractions from step 2.19 on an SDS-PAGE and Coomassie stain for analysis. NOTE: It is advisable to run an SDS-PAGE of samples from steps 2.9, 2.15 and 2.16 prior to gel filtration to confirm expression of the target protein.

## Representative Results

Here we show a 2 L (8 x 250 ml cultures) transient co-transfection and purification of the HDAC1, SDS3, Sin3A ternary complex. HDAC1 and SDS3 interact with Sin3A through the HDAC-interaction domain (HID) of Sin3A. A typical purification yields up to 1 mg of complex per liter of culture.


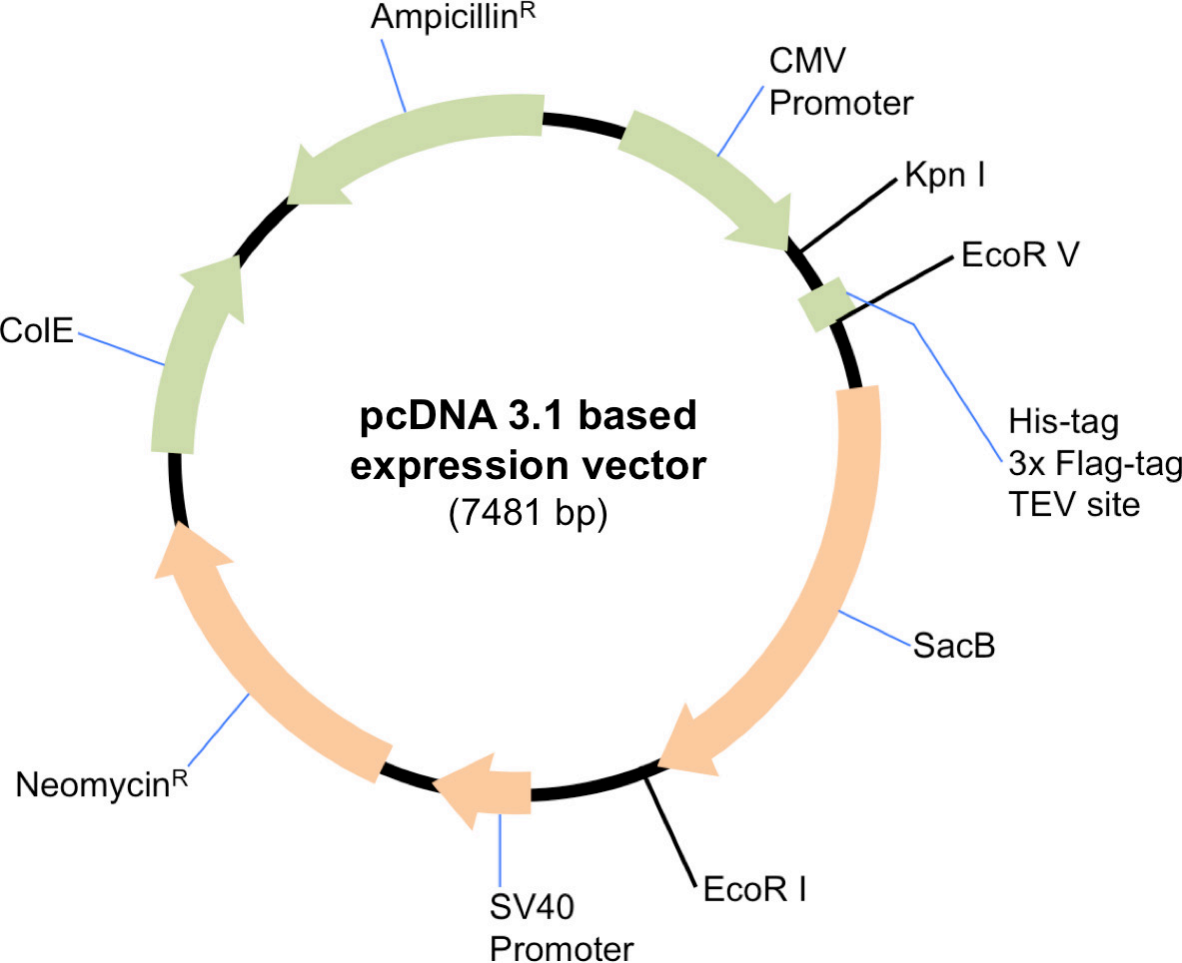
**Figure 1. Schematic of the modified pcDNA 3.1 expression vector.** The plasmid was digested with EcoRV and EcoRI to include the affinity-tag and TEV cleavage site on the amino terminus of Sin3A. To clone untagged versions of HDAC1 and SDS3, the vector was digested with KpnI and EcoRI.


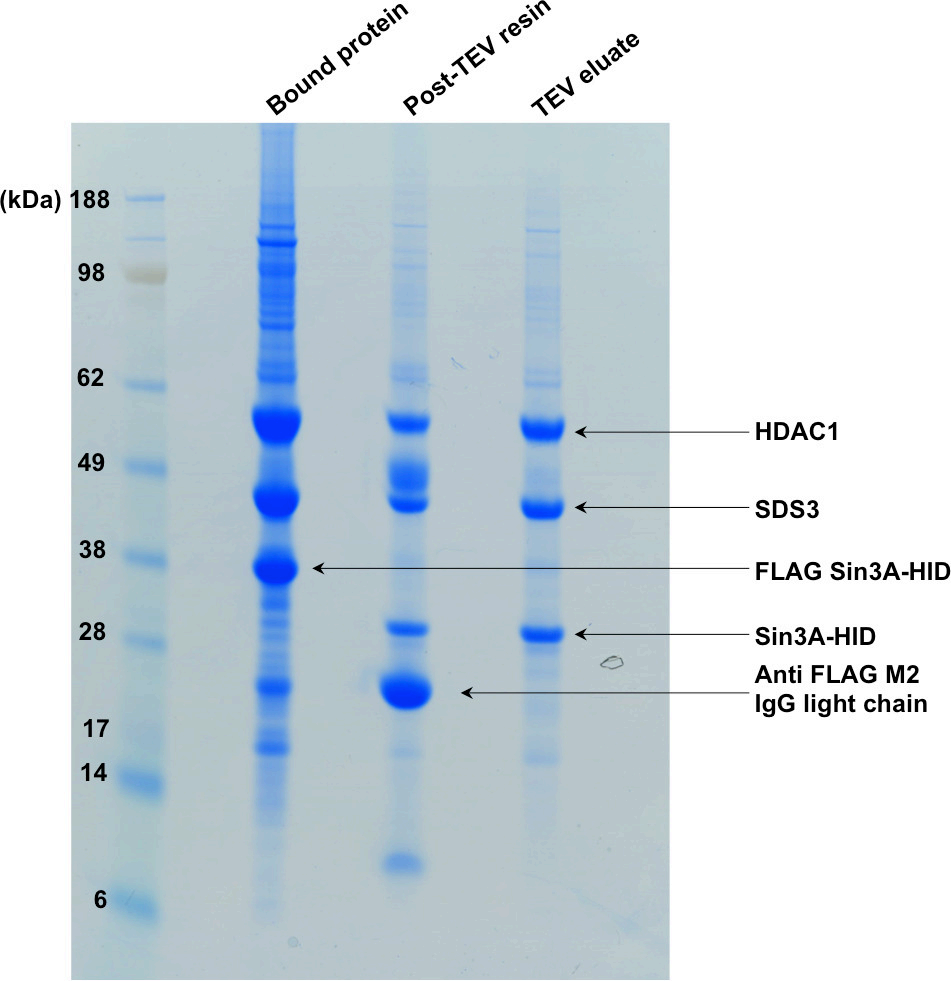
**Figure 2. SDS-PAGE showing the first step of the purification. **The “bound protein” lane shows the complex bound to the affinity resin. Following TEV digestion, the tag present on Sin3A-HID is cleaved off and the complex is eluted from the resin as shown in the second and third lanes of the gel.


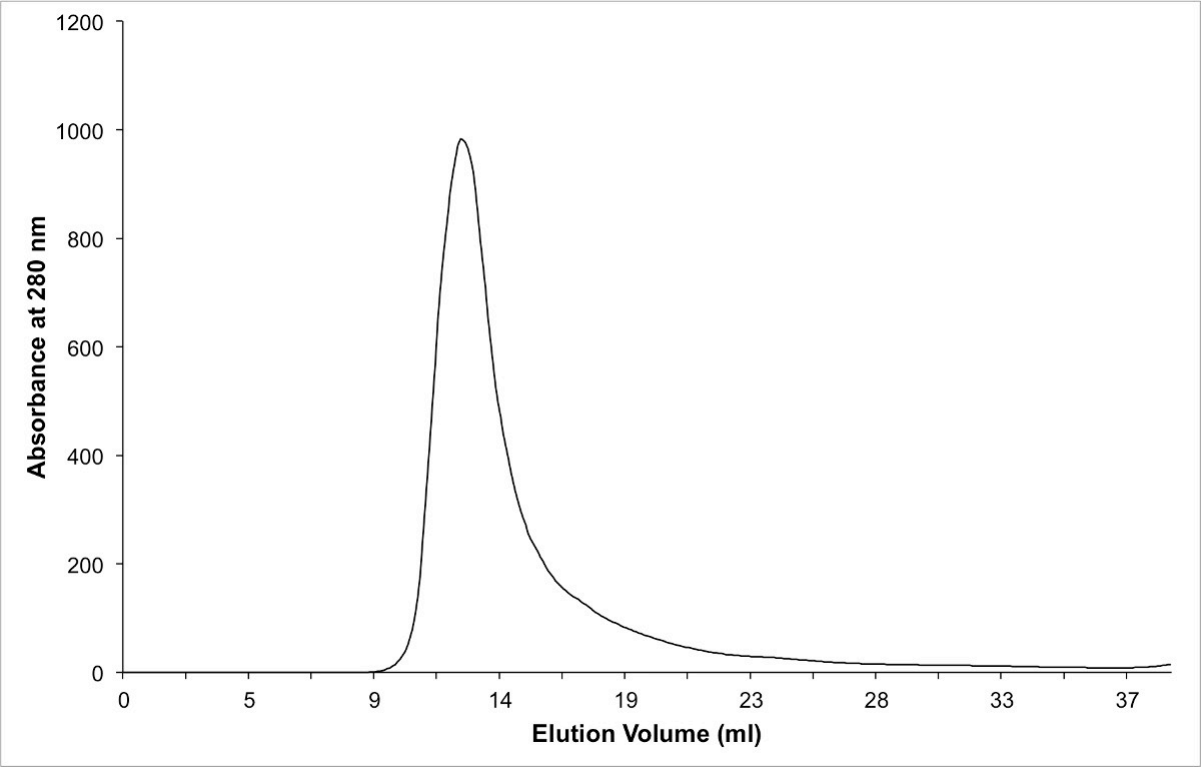
**Figure 3. Chromatogram of protein-complex purified using size exclusion chromatography.** Note that the pure complex eluted in the void volume because it forms a dimer in solution.


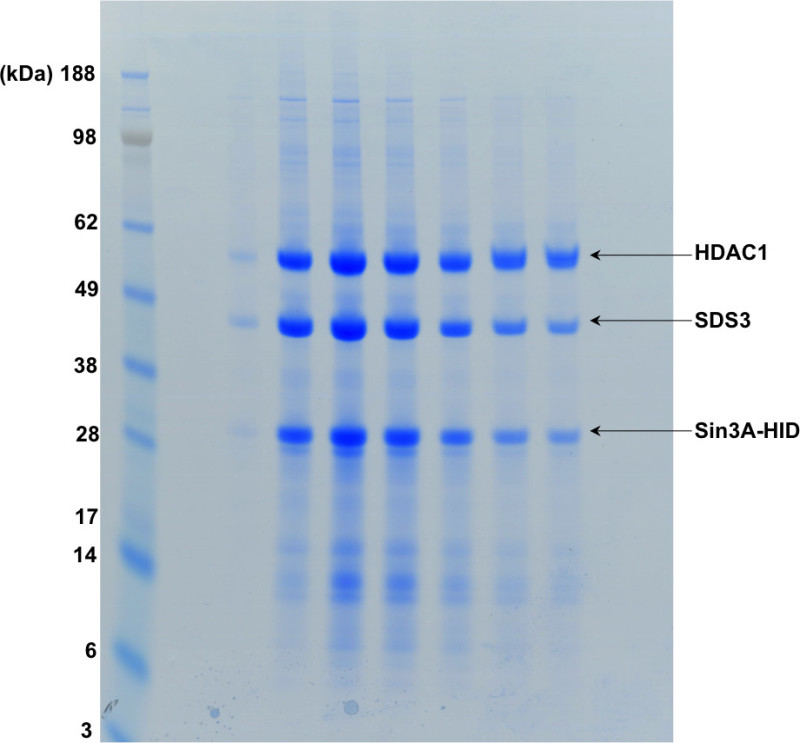
 **Figure 4. SDS-PAGE showing the fractions of the gel filtration shown in Figure 3.**


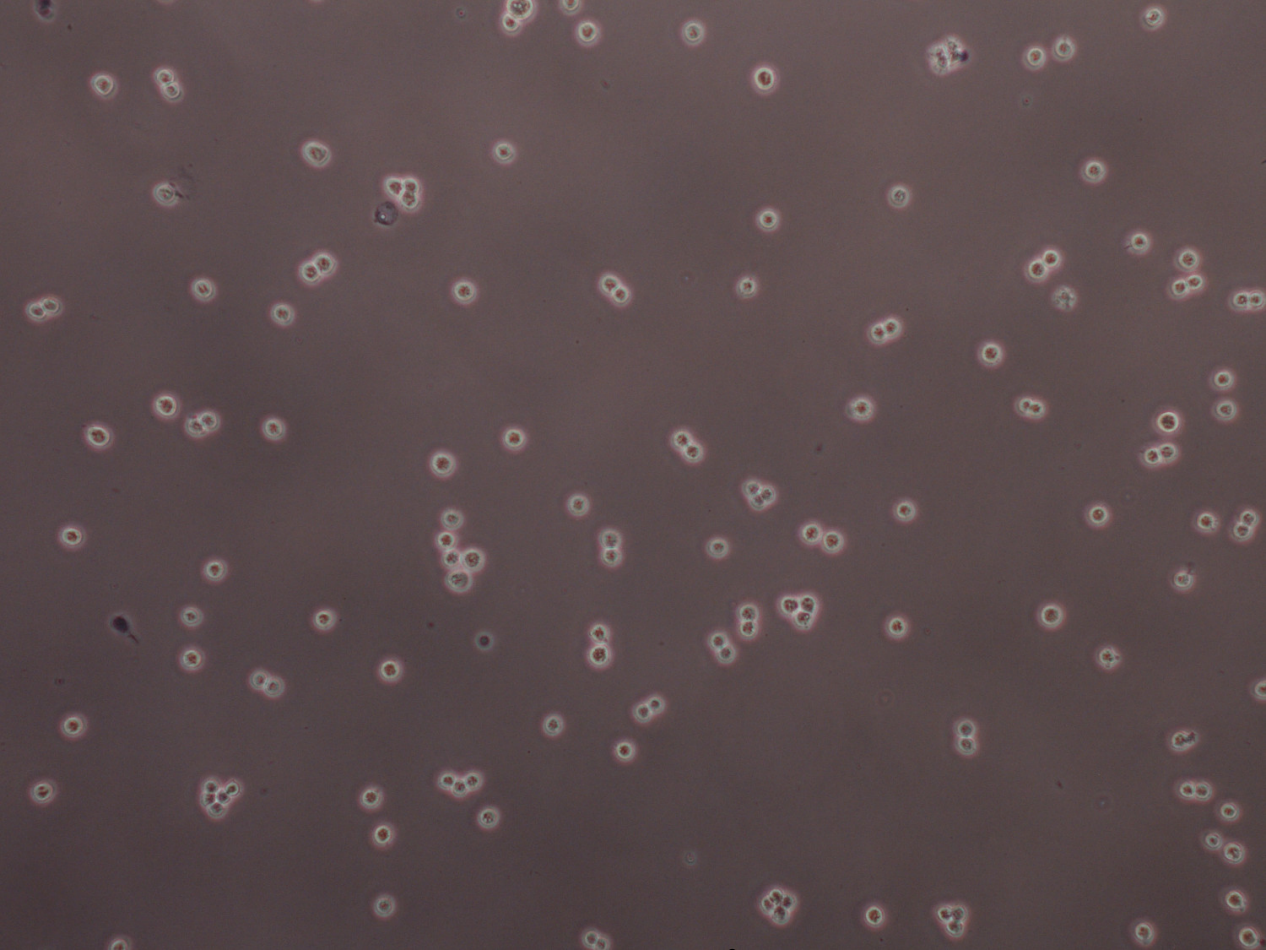
**Figure 5. Trypan blue-stained HEK 293F cells at 2.3x10^6 ^cells/ml ready to be transfected.** Cells should only be present as single or dividing cells, while large clusters may be broken up by vigorous vortexing for approximately 25 sec.

**Table:** 

**Problem**	**Possible causes**	**Action**
Low protein yield.	Low number of cells transfected.	Make sure cell density is approximately 1.0 x 10^6^ before adding the transfection reaction mixture to the culture.
Cells might have been subcultured too many times.	Use fresh stock cells after approximately 90 passages.
DNA may be degraded or have a high amount of impurities.	Make sure the plasmid DNA being used has a 260/280 ratio between 1.8 and 2.0. Running the DNA on an agarose gel is advisable to assess its quality.
Protein(s) being expressed are not stable or soluble enough.	Test different constructs in small scale prior to up-scaling the transfections. Make sure tags do not interfere with the structure of the protein(s) of interest.
Cells look cloudy and have an unusual color and/or odor.	Cells are infected with bacteria or yeast.	Good sterile technique must be used at all times. Fumigating the laminar flow hoods and UV-disinfecting the cell culture room in case of infection helps containing the problem.
Cells have low viability.	Wrong pH in media.	Make sure cultures are grown at 5-8% CO_2 _at all times.
Cells have been cultured up to a density over 3.0x10^6 ^ cells/ml.	Cells should not be grown up to densities above 2.5 x 10^6 ^cells/ml immediately before a transfection and never above 3.0 x 10^6 ^cells/ml.
Affinity purification did not work	Protein(s) is not being expressed.	See above.
Purification conditions might be wrong.	Adjust buffer conditions (*e.g.,* high salt, low salt, pH.)

**Table 1. Troubleshooting.**

**Table:** 

**System**	**Advantages**	**Disadvantages**
***E. coli***	Fast expression system High protein yields Inexpensive Suitable for the affordable production of isotopically labeled proteins	Lack of important chaperones that may be required for protein folding Lack of many post translational modifications May lack co-factors that are important for protein function and/or complex assembly.
***P. pastoris***	Can perform the majority of post translational modifications Inexpensive	Lack some post-translational modifications May lack co-factors that are important for protein function and/or complex assembly.
**Baculovirus expression in insect cells**	Can perform all post translational modifications Baculovirus is less cytotoxic than other viral strains	Preparing the virus for the transduction is time consuming The virus is not stable and cannot be stored for long periods of time Insect cells may lack co-factors that are important for protein function and/or complex assembly.
**Bioreactors with mammalian systems**	Can perform all post translational modifications Can provide all co-factors needed for protein function and complex assembly Can culture cells at very high densities Can express all mammalian proteins that require complex folding machineries Very high protein yields	Requires specialized staff to use the bioreactor. Expensive equipment is required. Optimizing expression conditions may be time consuming.
**HEK 293F suspension cells**	Can perform all post translational modifications Can provide all co-factors needed for protein function and complex assembly Can express all mammalian proteins that require complex folding machinery Fast and intuitive transfection protocol Relatively inexpensive compared to bioreactors and/or other commercially available transfection reagents.	Although affordable, not as cheap as expression in bacteria

**Table 2. Advantages and disadvantages of main expression systems.**

## Discussion

We have developed a straightforward and cost effective method (PEI costs much less than commercially available lipophilic transfection reagents) for expressing and purifying large amounts of recombinant proteins and multi-subunit complexes from mammalian cells. Optimal transfection and expression efficiency can be reached if highly pure plasmid DNA (260/280 between 1.8 and 2.0) is used in combination with PEI as described in the protocol section. Cells must be cultured in serum- and antibiotic-free media, therefore, sterile technique is strictly required for passaging and transfecting cells in order to avoid costly and time-consuming infections. Cell viability should be 90% or higher and cultures should not be grown to densities greater than 2.5 x 10^6 ^cells/ml immediately before a transfection, as doing so will reduce protein yield. For a successful transfection, cultures should only contain single or dividing cells. Clusters may be broken up by vigorous vortexing 20 to 30 sec (**Figure 5**). The ratio of each plasmid used in the co-transfection can be varied by the user according to the stoichiometry of the complex being studied. Expression efficiency can be optimized for the protein of interest, so that a suitable expression time can be established. Purification should be performed keeping the protein sample cold at all times to reduce the risk of unwanted proteolytic degradation. The choice of tags and purification buffers is critical, since they may interfere with structural elements or active sites of certain proteins, often resulting in reduced solubility and/or loss of enzymatic activity. Small scale transfections are particularly useful for testing different constructs for the purification of large protein complexes.

Today, a large variety of alternative methodologies are available for the expression of recombinant eukaryotic proteins (**Table 2**). For example, Baculovirus expression in insect cells is widely used due to its high transduction efficiency and its lack of cytotoxicity in comparison to other viral species^6^. However, the process of making the virus is time-consuming and its instability does not allow the virus to be stored for long periods. On the other hand, yeast expression systems offer the possibility of growing cells to very high densities in fermenter cultures, resulting in high protein yields^7^. But they still lack the full variety of post-translational modifications required for eukaryotic proteins to fold correctly and interact with their protein partners. HDAC3, for example, is expressed but does not fold in *E. coli*. However, when expressed in 293F cells, we were able to purify an active enzyme in complex with its co-repressor (SMRT) and a molecule of inositol tetraphosphate (IP4)^8^, which is not found in prokaryotic cells. This method has also allowed us to purify and solve the crystal structure of HDAC1 interacting with MTA1 in the NuRD complex, which is similarly activated by IP4^9^. Ideally, we would always like to express eukaryotic proteins in their natural, physiological environment. Mammalian expression systems employing HEK 293-EBNA1 cells in bioreactors^10-12^ have been described and yield very high levels of protein, but these can be complex to use.

Our method is a simple and accessible alternative to expression in bacteria, yeast and bioreactor systems. The expression method is fast and does not require the use of expensive equipment, particularly if the roller bottle or flask atmosphere is replaced with 5% CO_2 _and standard shaking incubators are used. We have used these protocols to co-transfect multiple plasmids and purify complexes with up to five proteins with yields of >1 mg/L of culture. Interestingly, the system helps the identification of stable well-behaved complexes. For instance, expression of the binary complex of HDAC1 and Sin3A gave limited yields of protein, yet addition of SDS3 resulted in 5-fold higher amounts and hence guides the structural biologist in choice of suitable constructs and stable complexes for crystallization.

## Disclosures

The authors declare that they have no competing financial interests.

## References

[B0] Baneyx F (1999). Recombinant protein expression in Escherichia coli. Current opinion in biotechnology.

[B1] Aricescu AR, Owens RJ (2013). Expression of recombinant glycoproteins in mammalian cells: towards an integrative approach to structural biology. Current opinion in structural biology.

[B2] Kim TK, Eberwine JH (2010). Mammalian cell transfection: the present and the future. Analytical and bioanalytical chemistry.

[B3] Boussif O, Zanta MA, Behr JP (1996). Optimized galenics improve in vitro gene transfer with cationic molecules up to 1000-fold. Gene therapy.

[B4] Godbey WT, Wu KK, Mikos AG (1999). Poly (ethylenimine) and its role in gene delivery. Journal of Controlled Release.

[B5] Beljelarskaya SN (2011). Baculovirus expression systems for production of recombinant proteins in insect and mammalian cells. Molecular Biology.

[B6] Cregg JM, Vedvick TS, Raschke WC (1993). Recent advances in the expression of foreign genes in Pichia pastoris. Nature Biotechnology.

[B7] Watson PJ, Fairall L, Santos GM, Schwabe JWR (2013). Structure of HDAC3 bound to co-repressor and inositol tetraphosphate. Nature.

[B8] Millard CJ, Watson PJ (2013). Class I HDACs Share a Common Mechanism of Regulation by Inositol Phosphates. Molecular Cell.

[B9] Tom R, Bisson L, Durocher Y (2008). Transfection of HEK293-EBNA1 Cells in Suspension with Linear PEI for Production of Recombinant Proteins. CSH protocols.

[B10] Durocher Y, Perret S, Kamen A (2002). High-level and high-throughput recombinant protein production by transient transfection of suspension-growing human 293-EBNA1 cells. Nucleic Acids Research.

[B11] Baldi L, Muller N (2005). Transient Gene Expression in Suspension HEK‐293 Cells: Application to Large‐Scale Protein Production. Biotechnology Progress.

